# Impact of physical activity on healthcare costs: a systematic review

**DOI:** 10.1186/s12913-023-09556-8

**Published:** 2023-06-03

**Authors:** Marjolein Duijvestijn, G. Ardine de Wit, Paul F. van Gils, G. C. Wanda Wendel-Vos

**Affiliations:** 1 National Institute for Public Health and the Environment, Bilthoven, The Netherlands; 2 Julius Center for Health Sciences and Primary Care, University Medical Center Utrecht, Utrecht, The Netherlands

**Keywords:** Physical inactivity, Healthcare costs, Physical-activity-related injuries, Systematic review

## Abstract

**Background:**

This systematic review aims to describe the relation between physical inactivity and healthcare costs, by taking into account healthcare costs of physical-inactivity-related diseases (common practice), including physical-activity-related injuries (new) and costs in life-years gained due to avoiding diseases (new), whenever available. Moreover, the association between physical inactivity and healthcare costs may both be negatively and positively impacted by increased physical activity.

**Methods:**

A systematic review was conducted, including records reporting on physical (in)activity in relation to healthcare costs for a general population. Studies were required to report sufficient information to calculate the percentage of total healthcare costs potentially attributable to physical inactivity.

**Results:**

Of the 264 records identified, 25 were included in this review. Included studies showed substantial variation in the assessment methods of physical activity and in type of costs included. Overall, studies showed that physical inactivity is related to higher healthcare costs. Only one study included costs of healthcare resources used in prolonged life when physical-inactivity-related diseases were averted, showing net higher healthcare costs. No study included healthcare costs for physical-activity-related injuries.

**Conclusions:**

Physical inactivity is associated with higher healthcare costs in the general population in the short-term. However, in the long-term aversion of diseases related with physical inactivity may increase longevity and, as a consequence, healthcare costs in life-years gained. Future studies should use a broad definition of costs, including costs in life-years gained and costs related to physical-activity-related injuries.

**Supplementary Information:**

The online version contains supplementary material available at 10.1186/s12913-023-09556-8.

## Background

World-wide, 28% of the population was insufficiently physically active in 2016 and this figure has not changed since 2001 [1]. According to the global physical activity guidelines of the World Health Organization (WHO), adults should engage in at least 150 min of moderate-intensity aerobic physical activity per week, or 75 min of vigorous-intensity aerobic activity, or an equivalent combination [2]. Not adhering to these recommendations increases the risk of cardiovascular diseases, stroke, type 2 diabetes, breast- and colon cancer [3, 4]. In addition, performing at least some physical activity has already been associated with improved health outcomes [5]. Next to averting disease, physical activity contributes to other aspects of health, for example a person’s well-being and quality of life [6, 7].

At the same time, healthcare costs are rising due to new technological possibilities, including expensive pharmaceuticals, increases in wages and prices and an ageing population. One other element in rising healthcare costs may be that unhealthy lifestyles, such as physical inactivity, may be associated with increased healthcare expenses. One global study showed the economic burden of physical inactivity to be substantial, especially in Western countries [8]. The impact of physical inactivity on healthcare costs may be twofold. On the one hand, when physical activity levels increase, physical-inactivity-related disease incidence declines, with associated lowering effects on healthcare costs [9]. A review by Ding et al. (2017) focused on this aspect when assessing the economic burden of physical inactivity [9]. On the other hand, reduced incidence of diseases associated with inactivity may extend people’s lives. As was shown in studies on obesity and smoking, improved lifestyle indeed reduced healthcare costs of diseases related to unhealthy lifestyles, but increased costs for unrelated diseases at the same time [10, 11]. A similar effect on healthcare costs of prolonged life could exist when physical inactivity would be reduced. For example, when living longer chances increase to ever need expensive nursing home care due to Alzheimer disease. Therefore, costs of healthcare resources used later in life should be taken into account in (reviews of) costing studies [12–15]. Moreover, improving physical activity levels could be accompanied with an increase in related injuries and contribute to healthcare costs as well [16]. These costs of physical activity related injuries were ignored in existing reviews on the relationship between physical inactivity and healthcare expenses. Hence, the association between physical inactivity and healthcare costs is not straightforward and may both be negatively and positively impacted by increased physical activity.

The aim of this systematic review is to describe the association between physical activity and healthcare costs, by not only taking into account healthcare costs of physical-inactivity-related diseases, but also extend to physical-activity-related injuries and healthcare resources used in life-years gained. The combination of these three aspects of healthcare costs related to physical activity has not been investigated before in a systematic review.

## Methods

### Data sources and search strategy

Peer-reviewed articles written in English or Dutch and published between 2010 and April 2020 were identified through both the EMBASE database and in references of eligible articles. The search strategy consisted primarily of two elements: ‘healthcare costs’ and ‘(in)physical activity’. Synonyms and related terms were used for both elements. The complete search string can be found in Supplementary Material 1. In addition to peer-reviewed articles, other reports or studies from the database of the Dutch Institute of Public Health and the Environment and in reference of eligible articles (reports not published in scientific journals), written in English or Dutch, were included as grey-literature. The reporting of this systematic review adheres to the Preferred Reporting Items for Systematic Reviews and Meta-Analyses (PRISMA) guidelines [17].

### In- and exclusion criteria

A set of inclusion criteria were applied to select publications eligible for this systematic review:

I. Studies needed to clearly state their definition of being (in)active;
II. Healthcare costs needed to be reported in such a way that they could be related to groups with different levels of physical (in)activity;
III. To enable comparison between studies with different methodologies, sufficient information needed to be available to calculate the percentage of total healthcare costs potentially attributable to physical inactivity;
IV. The study population needed to be representative of a national/geographical population or subgroup based on age or sex (e.g. study population did not consist of only people with a chronic disease);
V. Studies required a minimum reasonable sample size of at least 400 individuals.

Studies were excluded when a publication contained no original data (e.g. reviews and editorials) or were solely based on occupational physical activity. The latter because previous literature has indicated that the effect of occupational physical activity on health could oppose the effect of physical activity during leisure time [18].

### Study selection

**Fig. 1:**
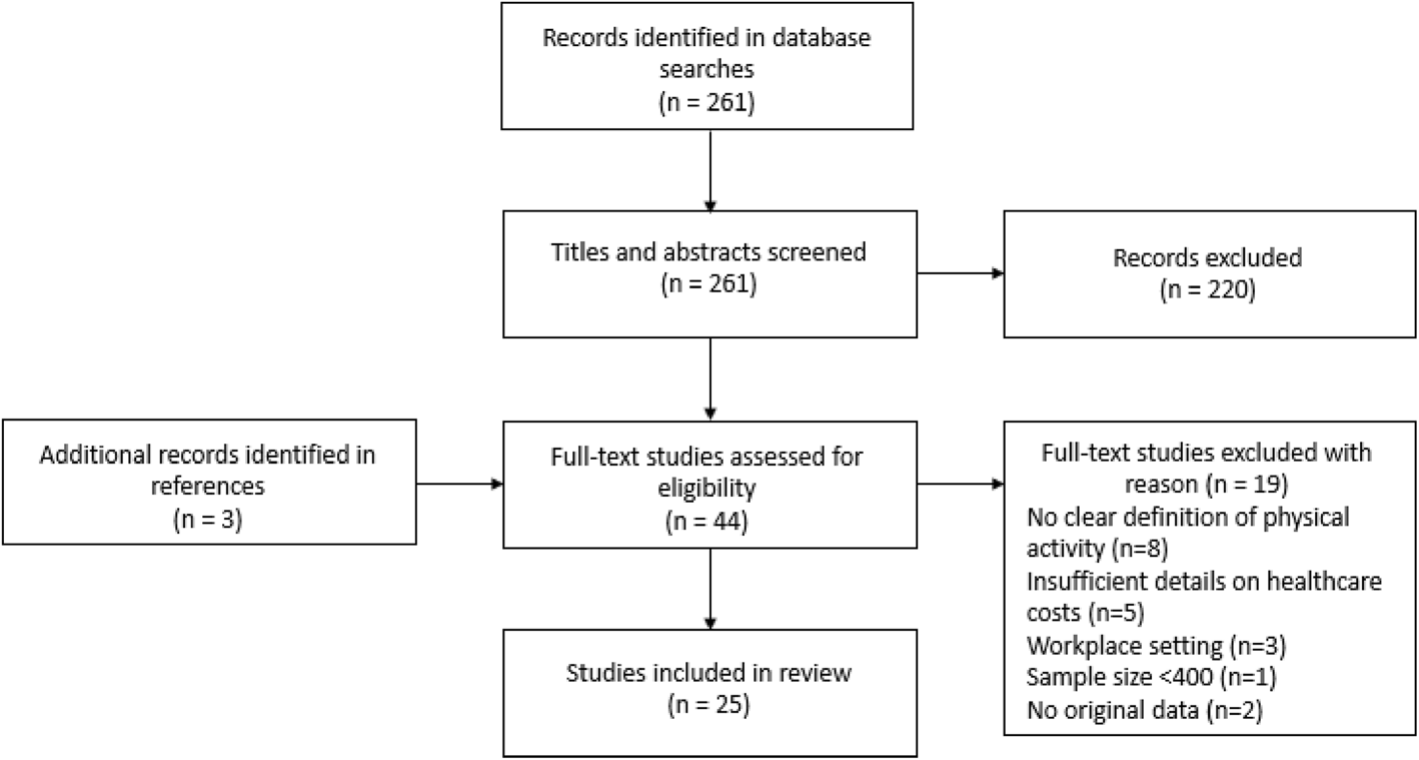
Flowchart of selection process

Titles and abstracts were screened for inclusion by two independent reviewers (MD, PvG). If at least one reviewer decided to include a record, the record was obtained for further screening. Full-text studies were also screened by two independent reviewers (MD, PvG) and consensus between the reviewers was required for a study to be included or excluded. A summary of the selection process is visualized in Fig. 1.

### Data extraction

Two reviewers (MD, PvG) highlighted relevant information in the full-text for data extraction. Data extraction was performed by one reviewer (MD) and verified by another (PvG).

Comparable to a previous review on the economic burden of physical activity [9], two methodological approaches were distinguished that describe the association between physical inactivity and healthcare costs. First, an econometric approach: data on physical inactivity and healthcare expenditure was combined at the individual level, thereby enabling a comparison of healthcare costs between physically active and inactive groups. Second, a population attributable fraction or PAF-based approach: healthcare costs attributable to physical inactivity were based on a combination of physical-inactivity-related health conditions and physical inactivity prevalence data [9]. With the PAF-based approach the proportion of disease that would not exist if physical inactivity was eliminated or reduced was estimated.

**Fig. 2:**
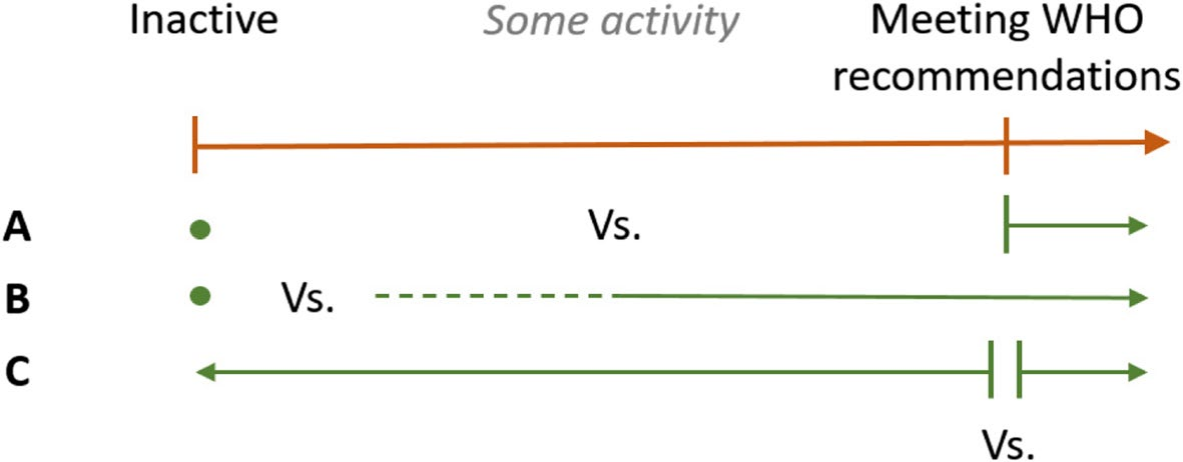
Categorization of studies to comparison **A**,** B** or **C** based on definition of sufficient physical activity applied. *WHO recommendations: engaging in at least 150 min of moderate-intensity physical activity per week and/or 75 min of vigorous-intensity activity*

With regard to physical activity, information on the definition of sufficient physical activity and the physical activity assessment tool was extracted. For PAF-based approach studies, the prevalence estimates of physical inactivity were extracted. Studies applied different definitions of being considered sufficiently physical active. These definitions were expressed in terms of time spent on moderate to vigorous intensity activities (e.g. ‘perform physical activity once a week’ or ‘adhere to WHO guidelines’) or based on energy expenditure in terms of calories per day. With respect to the wide range of definitions for physical (in)activity, comparisons were made between studies with similar definitions of sufficient physical activity. To do so, a predefined three-level scale was defined for level of comparison (Fig. 2), based on the WHO guidelines (150 min of at least moderate intense activity and/or 75 min of vigorous intense activity): A) ‘inactive vs. active according to the WHO guidelines’, B) ‘inactive vs. at least some physical activity’ or C) ‘inactive or at least some physical activity vs. adhering to the WHO guidelines’. By allocating the studies to the three comparison levels, individual study outcomes could be placed in perspective of the definition of sufficient physical activity used. A few econometric approach studies could be allocated to more than one category. For those studies, using data from one underlying population, results were not presented for all possible categories, to maintain equal weight of all studies in the aggregated results. In this case, results were presented for category B (see Fig. 2) as the majority of the econometric studies could be allocated to this comparison.

The outcome variable of interest for the included studies was healthcare costs. Extracted data included country, data source, time frame, healthcare costs and whether healthcare costs related to physical activity injuries were assessed. For studies with an econometric approach, additional methodological information was extracted on study design, sample size, age of population, adjustment for covariates and type of healthcare costs included. For PAF-based approach studies, additional information was extracted on health conditions included, use of crude risk ratios or adjusted risk ratios for confounders, correcting for comorbidities, applied scenario of becoming sufficiently physical active when calculating costs and whether healthcare costs related to improved life expectancy in the long-term was taken into account. For both types of studies the source of funding and possible conflict of interests were extracted as well.

### Data synthesis

Study characteristics of selected studies were summarized in an extraction sheet in Excel (Tables 1 and 2). The included characteristics were: country of the study, study sample, study design, reported comparison of physical activity (A, B, or C, see Fig. 2), type of healthcare costs included, time frame, confounders, diseases included and reported prevalence rate of physical inactivity. To compare study outcomes, the percentage of total healthcare costs attributable to physical inactivity was extracted. For PAF-based approach studies, this percentage was either extracted from the study directly, or calculated based on information available in the study (for example: (the difference in healthcare costs between inactive group and active group divided by healthcare costs of inactive group) × 100%)). For econometric approach studies, when the percentage was not mentioned it was calculated based on reported differences in healthcare costs between active and inactive groups (for example: (healthcare costs related to inactivity divided by total healthcare costs) × 100%).

**Table 1:** Characteristics of studies that applied an econometric approach (*n* = 14)

First author and reference	Country	Study design	Study sample	Confounders	Included healthcare costs	Time frame	Funding / Conflict of interest(COI)	Physical activity comparison category
Aljadhey 2012 [22]	United States	Cross-sectional	Adults aged ≥ 18 years (*n* = 270,553)	age, gender, race, insurance status, income, smoking, education, BMI	Office and hospital-based care, emergency room visits, inpatient hospital stays, dental visits, home health care, prescribed medications, vision aids, pharmaceutics	1 year (2002)	Funding not reported/ COI missing	B
Aoyagi et al. 2011 [23]	Japan	Cross-sectional	Communiity residents ageed ≥ 65 years (*n* = 5200)	Not reported	Public nursing care insurance costs and national health insurance expenditures of specific diseases, no inpatient costs	1 year (2009)	Funding reported/No COI declared	B
Carlson et al. 2015 [24]	United States	Cross-sectional	Adults aged ≥ 21 years (*n* = 51,165)	age, sex, race/ethnicity, marital status, census region, area, poverty, health insurance, education, smoking, BMI	Inpatient, outpatient, emergency room, office-based, dental, vision, home health, prescription drug, and other	1 year (2012)	Funding not reported/no COI declared	A
Chevan et al. 2014 [25]	United States	Cross-sectional	Adults aged ≥ 18 years (*n* = 8843)	age, sex, race, income, health status	All health services, drug expenditures, OOPC	1 year (2012)	Funding not reported/no COI declared	A,B,C
Dallmeyer et al. 2020 [26]	EU16 countries	Cross-sectional	Adults aged ≥ 50 years (*n* = 94,267)	age, gender, income, education, marital status, employment and immigration status, retired, children, household size, physical limitations, perceived health status, disease, smoking, overweight	OOPC for in- and outpatient care, drugs or home care	1 year (2013 and 2015)	Funding not reported/ COI missing	B
de Boer et al. 2020 [27]	Netherlands	Cross-sectional	99,8% of the Dutch population aged ≥ 19 years	Not reported	All basic health care; primary, hospital, pharmaceutical, mental health, dental and paramedical care	1 year (2016)	Funding reported/No COI declared	C
Kalbarczyk et al. 2019 [28]	Poland	Cross-sectional prospective	Adults aged ≥ 55 years (*n* = 1733)	age, sex	No information reported	47 years (2013–2060)	Funding reported/No COI declared	B
Kang et al. 2017 [29]	United States	Cross-sectional	Adults aged ≥ 18 years (*n* = 117,361)	age groups, sex, self-rated health, BMI, smoking, instrumental activities of daily living, serious psychological distress, poverty, health insurance, chronic diseases	Preventive, office-based, outpatient, inpatient, emergency department, home health, prescription medicines, OOPC	1 year (2007- 2011)	Funding reported/no COI declared	B
Karl et al. 2018 [30]	United Kingdom	Cross-sectional	Adults (age not reported)/ adults aged 48–68 years) (*n* = 2249/477)^a^	age, sex, income, smoking, alcohol, reporting on disease, problem walking	Physician visits, in- and outpatient hospital visits, pharmaceuticals	1 year (2013/ 2014)	Funding not reported/no COI declared	C
Min et al. 2016 [31]	South-Korea	Cohort	40 to 69 year old adults (*n* = 68,556)	age, gender, income, residential area, smoking, alcohol consumption, BMI	Inpatient, outpatient, and prescription costs	Several years 2005–2010	Funding reported/no COI declared	B
Peeters et al. 2014 [32]	Australia	Longitudinal	Women born in 1946–1951 (± 75 years old, *n* = 5535)	survey year, marital status, area of residence, education, smoking, BMI, depressive symptom	Subsidized health services by the government and OOPC, not public hospitals	1 year (2010)	Funding reported/no COI declared	A,B,C
Peeters et al. 2017 [33]	Australia	Longitudinal	Women aged 75.3 (1.5) years (*n* = 4678)	BMI, smoking status, depression and ability to walk 100 m	All healthcare costs paid by the government and OOPC, no inpatient hospital costs	1 year (1999–2013)	Funding reported/no COI declared	A,B,C
Sato et al. 2020 [34]	United States	Longitudinal observation	Adults aged ≥ 64 years (*n* = 611)	Not reported	Inpatient and outpatient care	3 years (2003–2014)	Funding not reported/ COI missing	B
Yang et al. 2011 [35]	Japan	Longitudinal	Adults aged ≥ 70 year (*n* = 483)	age, sex, hypertension, diseases, smoking, drinking, BMI, depressive symptoms, cognitive status	Inpatient and outpatient costs	5.5 years (2002–2008)	Funding reported/ COI missing	B

A (inactive vs. WHO guideline) B (inactive vs. at least some activity) C (inactive/insufficiently active vs. WHO guideline) ^a^physical activity measured with a questionnaire/measured with accelerometry,* OOPC* Out-of-pocket costs

**Table 2:** Characteristics of studies that applied a population attributable fraction (PAF) based approach (*n* = 11)

First author and reference	Country	Population sample	Included diseases	Funding /Conflict of interest (COI)	Reported prevalence of physical inactivity (%)	Physical activity comparison category
Amarasinghe 2010 [36]	Australia	Western Australian citizens ≥ 18 years old	Cardiovascular disease, stroke, diabetes, colon- and breast cancer, depression	Funding reported/ no COI declared	No information available	B
Ding et al. 2016 [8]	142 countries	Citizens of 142 countries	Cardiovascular disease, stroke, diabetes, colon- and breast cancer	Funding not reported/ no COI declared	23	C
In’t Panhuis -Plasmans et al. 2012 [37]	*Netherlands*	*Dutch citizens* ≥ *20 years old*	*Cardiovascular disease, stroke, diabetes, colon- and breast cancer*	*Funding not reported/ COI missing*	*No information available*	*C*
ISCA/Cebr 2015 [38]	*EU28 countries*	*European citizens of 28 countries*	*Cardiovascular disease, diabetes, colon- and breast cancer*	*Funding reported/ COI missing*	*26*	*C*
Janssen 2012 [39]	Canada	Canadian citizens ≥ 20 years old	Cardiovascular disease, stroke, diabetes, colon- and breast cancer, hypertension, osteoporosis	Funding reported/ COI missing	84	C
Krueger et al. 2015 [40]	Canada	Canadian citizens	Cardiovascular disease stroke, diabetes, colon- and breast cancer, hypertension, osteoporosis	Funding not reported/ COI missing	44	C
Krueger et al. 2016 [41]	Canada, BC	Citizens of British Columbia, Canada	Cardiovascular disease stroke, diabetes, colon- and breast cancer, hypertension, osteoporosis	Funding reported/ no COI declared	38	B
Mattli et al. 2019 [42]	Switserland	Citizens of Switzerland	Cardiovascular disease stroke, diabetes, colon- and breast cancer, hypertension, osteoporosis, depression, lower back pain	Funding not reported/ no COI declared	28^a^	B
Maresova 2014 [43]	Czech-Republic	Czech citizens ≥ 15 years old	Cardiovascular disease, stroke, diabetes, colon- and breast cancer	Funding reported/ COI missing	18	C
Market Economics Limited 2013 [44]	*New-Zealand*	*New-Zealand citizens* ≥ *25 years old*	*Cardiovascular disease stroke, diabetes, colon- and breast cancer, hypertension, osteoporosis, depression*	*Funding reported/ COI missing*	*48*	*C*
Scarborough et al. 2012 [45]	United Kingdom	Citizens of United Kingdom	Cardiovascular disease, stroke, diabetes, colon- and breast cancer	Funding reported/ COI missing	18	B

Grey literature in *italic*. A (inactive vs. WHO guideline) B (inactive vs. at least some activity) C (inactive/insufficiently active vs. WHO guideline) ^a^ number not mentioned in study but could be calculated based on available information

To facilitate comparison of healthcare costs between studies from different base years, estimates were inflated from the year of original data to 2019 using annual consumer price index as provided by Statistics Netherlands (CBS) [19]. Costs in local currencies and different years were converted to purchase power parity Euro’s (year 2019), using conversion factors provided by the Organization for Economic Co-operation and Development (OECD) [20].

### Quality assessment

The quality of studies was assessed with the “Checklist for reporting estimates of the economic costs/burden of risk factors” based on the “Consolidated Health Economic Evaluation Reporting Standards” (CHEERS) [21]. This checklist is the first tool with guidelines for studies that estimate the economic burden of risk factors [9]. The checklist consists of 19 items to assess the risk of bias. This instrument was chosen because it provides the opportunity to assess relevant risk of bias elements of healthcare costs studies and allows comparison of the methods of healthcare costing studies [9]. For each study information related to healthcare costs was scored, per item with ‘yes’, ‘no’ or ‘not applicable’.

## Results

### Selection of studies

The search in the EMBASE and RIVM database resulted in 261 records and three articles were found through references of eligible articles, 220 were excluded based on the applied inclusion criteria on the title or abstract, leaving 41 records for full-text inspection (Fig. 1). Additionally, three records were identified through references. Excluded articles often focused on groups with specific health conditions and did not relate to general population samples. After reading the full-text articles 19 publications were further excluded. Articles were mainly excluded because of an unclear definition of being considered physically active/inactive or because healthcare costs were not related to physical (in)activity. In total, 25 publications were included (Fig. 1) of which 22 were peer-reviewed articles and 3 were grey literature reports. One of the 22 peer-reviewed articles and two grey literature reports were included based on the reference list of included articles.

### Study characteristics

Of the 25 studies included, 14 studies used an econometric approach and 11 studies a PAF-based approach. Studies were conducted in Europe (9 studies), North-America (8 studies), Australia (4 studies), and Asia (3 studies) while one study had a world-wide scope. For econometric studies, the sample size ranged from 483 to 117,361 individuals. Half of the studies reported on a senior population over 50 years of age, of which five studies reported on a population of 65 years old and above. Half of the PAF-based studies reported on a population irrespective of age, the other half reported on populations from the age of 15, 18, 20 or 25 years old (Tables 1 and 2).

In general, the level of physical activity was based on questionnaires. The type of questionnaire showed large variety between studies. Only three studies reported to have used a validated questionnaire [27, 34, 43]. Two studies measured physical activity with an accelerometer [30, 39]. In most studies, physically active individuals were classified according to the WHO guideline and could be allocated to comparison group A or C (Table 1). In a few studies, individuals were regarded as active when performing at least some activity. Following the protocol for this systematic review, these studies were allocated to comparison group B (Fig. 2).

Studies with an econometric approach used a broad range of healthcare expenditures, based on the available data source (Table 1). In most studies, healthcare cost data was extracted from healthcare registries, based on health insurance claims, or from national healthcare system databases. Some studies used self-reported healthcare expenditure data [26, 29, 30]. Moreover, PAF-based approach studies considered healthcare cost for treating a range of health conditions. Nearly all studies included cardiovascular diseases, stroke, type 2 diabetes, breast- and colon cancer (Table 2). Half of the studies also included osteoporosis and hypertension [23, 36, 39, 40, 42, 44, 46] and assessed other health conditions such as depression [23, 36, 42, 44] and lower backpain [42] (Table 2).

### Costs of physical inactivity

All studies, except one [30], confirmed that physical inactivity is related to higher healthcare costs (Table 3). The one exception study, Karl et al. (2018), did find a relation between time spent on physical inactivity and healthcare costs when physical activity was assessed with an accelerometer, however, when using WHO guidelines as a cut-off no significant difference was found between active and inactive groups [30]. For the studies with different definitions of sufficient physical activity, no clear differences in outcome measure could be identified (e.g. in Table 3 percentage of total healthcare costs related to physical inactivity in category A, B, or C show no consistent results).

**Table 3:** Study outcomes estimating healthcare costs of physical inactivity per year and quality assessment (*n* = 25)

	Percentage of total healthcare costs related to physical inactivity in physical activity comparison category (%)			Lower healthcare costs for active groups (€, per person)	Score items on checklist(#yes/ #applicable items) [9]
**Econometric approach**	A	B	C		
Aljadhey 2012 [22]		16.7^a c^		846	13/18
Aoyagi et al. 2011 [23]		3.7 (+ 5% PA)		126	15/17
Carlson et al. 2015 [24]	26.6			1340	15/18
Chevan et al. 2014 [25]		25.0^a b^		2431^a b^	14/18
Dallmeyer et al. 2020 [26]		14.1^a^		42^a^	17/18
de Boer et al. 2020 [27]			0.6 (+ 1% PA)	15	16/18
Kalbarczyk et al. 2019 [28]		0.4–1.6^d^		No information reported	16/18
Kang et al. 2017 [29]		11.1^a^		453^a^	14/18
Karl et al. 2018 [30]			None	None	16/18
Min et al. 2016 [31]		11.7^a^		106^a^	15/18
Peeters et al. 2014 [32]		9.0^a^		44^a^	16/18
Peeters et al. 2017 [33]		22.2^a c^		247^a^	15/18
Sato et al. 2020 [34]		0.4 (lag 2 years) 1.0 (lag 3 years) (+ 10% PA)		No information reported	14/19
Yang et al. 2011 [35]		15.5^a^		134^a^	15/18
	Percentage of total healthcare costs related to physical inactivity in physical activity comparison category (%)			Healthcare costs attributed to physical inactivity (€, million)	Score items on checklist(#yes/ #applicable items) [9]
**PAF based approach**	A	B	C		
Amarasinghe 2010 [36]			0.9^a^ (+ 10% PA)	4.4	17/18
Ding et al. 2016 [8]			0.6	46,268.0	17/18
In’t Panhuis -Plasmans et al. 2012 [37]			1.8	1542.2	16/18
ISCA/Cebr 2015 [38]			0.7^a^	10,212.0	16/18
Janssen 2012 [39]			3.8	2026.0	16/18
Krueger et al. 2015 [40]		1.5^a^		2291.1	16/18
Krueger et al. 2016 [41]		1.1^a^		244.7	17/18
Maresova 2014 [43]			0.4	48.5	15/18
Market Economics Limited 2013 [44]			4.6	405.2	17/18
Mattli et al. 2019 [42]			1.2	6.3^a^	17/18
Scarborough et al. 2012 [45]		1.2^a^		1378.9	16/18

Grey literature in italic. A (inactive vs. WHO guideline) B (inactive vs. at least some activity) C (inactive/some activity vs. WHO guideline), *PA * Physical activity

^a^figure not mentioned in study but could be derived from available information

^b^no difference when correcting for covariates

^c^number without correcting for confounders

^d^percentage of Gross Domestic Product instead of total healthcare costs. Costs were converted to PPP (2019 €)

Nine econometric studies comparing physically active and inactive groups showed lower healthcare costs for the active groups, ranging from 9.0% to 26.6% lower costs [22, 24–26, 29, 31–33, 35]. One of these studies found no association between physical inactivity and healthcare costs when perceived health was taking into account as a covariate [25]. Next to that, three econometric studies calculated healthcare costs related to an extra proportion of the population becoming sufficiently active [23, 27, 34] and one estimated costs as percentage of the country’s GDP [28]. These four studies also showed that a proportion of total healthcare costs can be attributed to physical inactivity, the studies will be described one by one. De Boer et al. (2020) calculated the percentage of total healthcare costs at national level that could be saved if an extra percentage of the population would adhere to WHO guidelines. They estimated that with each 1% increase in adherence to the guidelines, 0.4% of the total population’s healthcare costs could be saved [27]. Aoyagi et al. (2011) used a similar approach. Instead of using the difference in healthcare costs between groups they used healthcare expenditure data based on health conditions related to physical inactivity. They concluded that if 5% of the currently inactive population would become sufficiently active, 3.7% of healthcare expenditure on health conditions related to physical inactivity could be averted [23]. Sato et al. (2020) were the only ones to report on the healthcare costs with a time lag between risk factor and healthcare costs, they concluded that if 10% of the population would become sufficiently active, after 2 years 0.4% and after 3 years 1.0% of total healthcare cost could be averted [34]. Covariates adjusted for in econometric studies varied (Table 1).

PAF-based approach studies showed that 0.4% to 4.6% of total healthcare costs could be attributed to physical inactivity [8, 36–45]. In these studies, a scenario was applied that the whole population would need to become sufficiently active to avert the calculated costs. One of these studies, Amarasinghe (2010), assessed a scenario in which 10% of the population would become more active, resulting in 0.9% less healthcare expenditure [36]. Difference in study findings can be attributed to differences in included health conditions, physical activity definition and in physical inactivity prevalence rates. One study adjusted for possible comorbidities [44]. The majority of the studies used crude risk ratios in the calculation and several studies applied adjusted risk ratios for confounders [8, 37, 39–41]. No clear distinction in healthcare costs was found between these two methodological approaches. A small difference in costs is seen between the five studies including four to five common health conditions (0.4%-1.8% of healthcare costs attributable to physical inactivity) [8, 37, 38, 43, 45], and the studies including additional diseases, reporting percentages of 0.9%-4.6% of healthcare costs being attributable to physical inactivity [36, 39–42, 44].

### Cost of physical activity related injuries

None of the studies included in our review combined healthcare costs related to physical- activity-injuries with healthcare costs of physical-inactivity-related diseases.

### Healthcare costs in life-years gained

Only one study considered the healthcare costs associated with additional life-years as a consequence of being more physically active [37]. In ‘t Panhuis-Plasmans (2012) estimated that 1.8% of total healthcare costs could be related to physical inactivity. Moreover, healthcare costs related to chronic diseases would decrease with 3% when the whole population would be sufficiently active, but at the same time increase with 4% when healthcare expenses in additional life-years were included [37]. Hence, the net effect on healthcare costs was found to be negative when the positive effect of physical activity on life-expectancy was included in the analysis.

### Quality assessment of studies included in our review

Studies reported ‘yes’ on 13 to 17 items out of 17, 18 or 19 applicable items of the “Checklist for reporting estimates of the economic costs/burden of risk factors” (Table 3). Eleven studies did not report on a sensitivity analysis (item 16) and nine studies did not elaborate on relevant aspects of the healthcare system in which decisions needed to be made (item 5). Seventeen studies reported costs separately for subgroups to characterize heterogeneity (item 17). The majority of the studies reported on the source of funding (item 18) and half of the studies on competing interests (item 19, for further details see Supplementary Material 2).

## Discussion

This study aimed to describe the association between physical activity and healthcare costs, by taking several aspects of healthcare costs related to physical activity into consideration. Next to including healthcare costs of diseases directly related to physical inactivity, costs of physical-activity-related injuries and healthcare costs in life years gained were included in this study, as advised by many health economists, and Dutch and US guidelines for costing studies [13–15]. Although the aim of the study was novel, this study revealed that most studies on the relationship between physical inactivity and healthcare costs adopted a limited perspective on healthcare costs, by only including healthcare costs directly relating to physical inactivity. Similar to previous literature our findings showed that physical activity could reduce physical-inactivity-related healthcare costs [9].

A strength of the study is the broader definition of healthcare costs related to physical inactivity. Although this broader definition, including costs in life years gained, has been adopted in other fields of disease prevention (e.g. obesity, smoking) [10, 11], this study is the first review to include these future medical costs in relation to physical inactivity. Indeed, the single study in this review that did include these future costs showed that the use of healthcare resources in increased life-years do increase overall cost estimates.

There are some limitations of the current review that should be considered. First, in the literature search a narrow time-frame of 10 years was used (2010–2019). The reason for this time-frame was to include the most recent literature (as over time previous studies were updated and studies often reported on healthcare costs of several years before publication date). Publications from before 2010 were considered to provide outdated information on healthcare costs. Nevertheless, a previous review by Ding et al. (2017) on the economic burden of physical inactivity that did include studies published before 2010 came to comparable conclusions for proportion of healthcare costs attributable to physical inactivity based on PAF based studies (0.3%-4.6% vs. 0.4%-4.6%) [9].

Second, the search was conducted in only one database, EMBASE, thereby possibly missing relevant studies. However, EMBASE is considered to be an up-to-date date source on biomedical research and covers the most important international biomedical literature. Next to that, the review of Ding et al. (2017) was screened for additional eligible articles. This revealed only one new peer-reviewed article and two grey literature publications that met our inclusion criteria (Fig. 1) [9].

Last, in this study information on physical activity and related healthcare costs was dichotomized by categorizing studies based on the study’s definition of sufficient physical activity. We acknowledge that some information could get lost following this approach. For example, for category B (inactive + some activity vs. WHO guideline) it implicitly is assumed that performing only some physical activity does not contribute to health [47]. Thereby, the presented healthcare costs might be an underestimation of the true effects of physical inactivity. Another reason for an underestimation is that savings or reductions in health costs associated with therapies derived from physical activity were not included in the current study. After all, physical activity has an inverse dose–response relationship with mortality [2, 48].

With an increase of the population’s physical activity level, it is likely that the incidence of physical-activity-related injuries increases as well (e.g. cycle injuries during active transport, sport injuries). Unfortunately, based on the inclusion criteria of this review no study could be included that mentioned to have considered costs related to physical activity injuries. A report that studied healthcare costs of sport injuries in relation to healthcare costs for physical-inactivity-related disease concluded that for adults aged 25 to 54 years costs for sport injuries (500–800 Euro per person) did not outweigh healthcare cost related to averting physical-inactivity-related diseases (1500–6000 Euro per person), showing that net healthcare costs are not higher for active individuals [49]. It should be acknowledged that some studies in our review implicitly included healthcare costs for physical-activity-related injuries as they compared all healthcare costs between inactive and active groups. These studies reported lower healthcare costs for physically active groups, despite the inclusion of physical-activity-related injuries. Moreover, physical-activity-related injuries can differ in seriousness and can have a short-term (e.g. sprain ankle) or long-term (e.g. arthrosis) character, with a varying impact on healthcare costs. To date, research on quantifying healthcare costs of physical-activity-related-injuries is limited. Therefore, more research is needed to include this aspect in calculations of healthcare costs related to physical activity.

A fair comparison of results between studies depends on the comparability of methods. Methods of studies included in this review vary, for example with regard to age group included, number and type of diseases included, type of healthcare costs included, data sources and definition of physical activity. In the current review, it was not possible to draw conclusions on whether differences between study findings could have been due to a variation in the applied definition of physical activity. Moreover, a variety of measurement tools was used to assess physical activity (e.g. validated and unvalidated question(naires), accelerometry). Furthermore, self-reported data is known to result in overestimation of physical activity [50]. Also, included studies often failed to correct for comorbidities which could result in an overestimation of healthcare costs. Additionally, overestimation could have been the case for several econometric approach studies included in this review. The study populations of several studies often consisted of elderly samples, for whom physical-inactivity-related diseases are more likely to occur. This illustrates the importance of proper reporting on methodological features, key assumptions and limitations, and to enable harmonization based on output. Similarly, as argued by Ding et al. (2017), future studies should preferably use a checklist for reporting estimates of the economic costs/burden of risk factors [9].

Findings in this review are mostly based on cross-sectional data and do not give information about long-term physical (in)activity behavior, which plays an important role in relation to healthcare costs. To fully understand this relation, information from a life-time perspective is required. Even though conducting longitudinal studies could be difficult and time demanding, it is needed to derive more accurate estimates of changes in physical activity behavior and subsequent changes in healthcare costs.

The current review focused on healthcare cost related to physical activity, ignoring wider societal costs, which are advised to include by many guidelines for health economic studies [13–15]. For instance, active individuals show less work absenteeism [51, 52]. In addition, active individuals may also contribute to informal caregiving more often than inactive persons. Moreover, from a societal perspective, physical activity positively contributes to a person’s well-being and quality of life [6, 7]. At the same time, productivity of active individuals may be impacted by sports injuries [53]. Therefore, total societal costs and benefits of enhanced physical activity could be larger than found in this study. Future health economic studies should preferably make an effort to adopt this broader societal perspective.

Results of this literature review may give insight in the net-effect of physical activity on healthcare costs, which is of importance for knowledge based public health policy. Moreover, the results could encourage public health policy to keep striving for a physically active population, as this may result in reduced healthcare costs in the short-term, next to apparent health effects of physical activity. These insights can add to the justification of policy decisions on (further) investments in physical activity and sports. Additionally, we encourage research institutes outside of academia to publish their study results more often in scientific journals, next to publishing standard reports’ in their mother tongue, thereby, enhancing the body of research in the field of physical inactivity and health economics.

## Conclusion

Similar to previous research, this review concluded that increasing physical activity is associated with lower healthcare costs in a general population in the short-term. Although studies were difficult to compare due to different methods, the majority of the studies showed physically active populations to have lower healthcare costs. However, this review is the first review to have included future medical costs, suggesting that healthcare costs in the long run may increase in a more physically active population. No studies that included costs of physical-activity-related injuries were found. Future research should focus on combining all healthcare costs, both positively and negatively associated with physical inactivity. In addition, studies may adopt a societal perspective instead of the healthcare perspective by including absenteeism costs related to physical inactivity and physical-activity-injuries. Thereby, future studies may provide a more comprehensive picture of economic benefits of physical activity.

## Supplementary Information

**Additional file 1:** **Supplementary Material 1. **Complete search string.

**Additional file 2:** **Supplementary Material 2**. Quality assessment.

## Data Availability

All data is available from the published articles and reports, see reference list.

## Abbreviation

PA: Physical activity
