# Supplementary material for: Impact of physical activity on healthcare costs: a systematic review
Source: BMC Health Serv Res. 2023 Jun 3;23:572. doi: 10.1186/s12913-023-09556-8 (PMC10239135; doi:10.1186/s12913-023-09556-8)
Supplement: Supplementary file 1 — Additional file 1: Supplementary Material 1. Complete search string. [file 12913_2023_9556_MOESM1_ESM.docx]

**Supplementary Material 1.** The complete search string.

#1 'health insurance' OR 'health insuran*'

OR

#2 'health care cost' OR 'healthcare cost*' OR 'health care cost*'

'healthcare expenditure*' OR 'health care expenditure*' OR 'health expenditure*'

OR

#3 ‘disease burden’ OR ‘economic aspect’ OR ‘economic burden’

AND

#4 'sport' OR 'sport*' OR 'physical activity, capacity and performance' AND 'review' OR 'physical inactivity' OR 'physical inactiv*' OR 'sport inactiv*' OR 'physical activity' OR 'physical activ*' OR 'sport activ*' OR 'sedentary lifestyle' OR 'sedentary lifestyl*' OR 'sedentary behavio*' OR 'sedentary lifestyle' OR 'sedentary lifestyl*' OR 'sedentary behavio*' OR 'exercise' OR 'exercis*' OR 'sport injury' OR 'sport injur*' OR 'light physical activity' OR 'light physical*' OR 'moderate physical activity' OR 'moderate physical*' OR 'vigorous physical activity' OR 'vigorous physical*' OR 'cycling' OR 'cycling*' OR 'bicycling*' OR 'walking' OR 'walking*' OR 'running' OR 'running*' OR 'athletics' OR 'athletic*' OR 'jogging' OR 'jogging*' OR 'winter sport' OR 'skiing* OR 'skating* OR 'ice hockey*’ OR 'skateboarding' OR 'skateboard*' OR 'racquet sport' OR 'tennis*' OR 'squash*' OR 'badminton*'

AND

#5 ‘article’ OR ‘article in press’ OR ‘editorial’ OR ‘letter’ OR ‘Note’ OR ‘review’ AND [2010-2020]
